# Effect of Hot-Pressing Temperature on Characteristics of Straw-Based Binderless Fiberboards with Pulping Effluent

**DOI:** 10.3390/ma12060922

**Published:** 2019-03-20

**Authors:** Jiajun Wang, Bo Wang, Junliang Liu, Lin Ni, Jianzhang Li

**Affiliations:** 1 Research Institute of Wood Industry, Chinese Academy of Forestry, Beijing 100091, China; wangjiajun@caf.ac.cn (J.W.); nilin@caf.ac.cn (L.N.); 2 MOE Key Laboratory of Wooden Material Science and Application, Beijing Key Laboratory of Wood Science and Engineering, MOE Engineering Research Centre of Forestry Biomass Materials and Bioenergy, Beijing Forestry University, Beijing 100083, China; 3 Tianjin Rongyeda Technology Development Co., Ltd., Tianjin 300384, China; wangbo0035@hotmail.com

**Keywords:** wheat straw, temperature, pulping effluent, self-bonding, fiberboards

## Abstract

This study aimed to improve straw-based fiberboard properties without resins by adding pulping effluent as well as to investigate the difference among boards under variable hot-pressing temperatures. The characterization of fiberboards produced from wheat straw under pressing temperatures ranging from 160 to 200 °C was first described. The surface appearance, surface chemistry, thermal transitions, and mechanical performance of the boards were evaluated to investigate the effect of varying hot-pressing temperature. The results indicated that the surface color of boards became darker when the temperature was above 190 °C. Additionally, Fourier transform infrared (FT-IR) measurements showed that more low-molecular constituents and hydrogen bonds were produced under higher pressing temperatures. Furthermore, the physical and mechanical property data were analyzed statistically using one-way analysis of variance (ANOVA) and Tukey’s tests (α = 0.05). The results demonstrated that straw-based fiberboards with effluent under 190 °C exhibited superior strength and water resistance capacities, and showed great potential in commercial decorating and packaging applications.

## 1. Introduction

Biomass has been brought into focus as a renewable resource in diversiform domains, which is promising for the reduction of carbon emissions and the promotion of cyclic utilization for non-renewable materials. Wheat straw (Triticum aestivum Linn.), which is one of the most abundant agricultural surpluses in the world, has the potential to facilitate price stabilization for sustainable economic development in rural areas. Approximately 175 million tons of dry wheat straw was collected in China in 2015 [1,2]; however, this abundant regenerative resource is not effectively utilized at present. Traditional uses of wheat straw include paper making, acidic sulfite pulping, fiberboard manufacturing, use as feed for animals, and soil conditioning [3,4,5,6]. The lack of waste management for this product, combined with the high amount of excess wheat straw, drives the needs for new and high-value applications of such residues. The problem is much more urgent in developing parts of the world, where the direct outdoor burning of superfluous wheat straw is an inevitable fire hazard and causes air pollution. 

As a natural material, wheat straw has been widely used for several decades in the production of fiber and its derivative products. Fiberboards, in combination with fibrous-felted and homogeneous panels, are used for interior door skins, moldings, flooring substrate, and interior trim components. Traditionally, fibers from non-renewable resources are bonded together with adhesives [7,8]. However, this process has a number of drawbacks, including the high cost of adhesives [9] and toxic formaldehyde emissions. Omitting the usage of resins in fiberboard manufacturing has the potential to be environmentally friendly and low-cost. Research has been conducted on the production of self-bonding boards using various raw materials [10], including woody and non-woody raw materials. Halvarsson [11] et al. described the straw fibers as a low-budget starting material for binder-free fiberboard. For the purpose of reinforcing fiber interlacing to a level of ideal performance, Fenton’s reagent [12], enzymatic additives [13,14], and other additives [15,16,17] have also been used for pretreating raw materials [9]. Combined with these additives, pressing temperature is one of the most important factors influencing panel characteristics. A higher pressing temperature results in the emission of extractives [18,19], and the mechanical properties of panels can be controlled by various temperature intervals. Chemical changes with temperature variations can contribute to self-bonding, and the reduction of water absorption also results from the degradation of hemicelluloses [20]. The physical-mechanical performance of binderless boards made from sugarcane bagasse were modified by increasing the pressing temperature according to Okuda [21]. Chow et al. [22] manufactured boards without an adhesion agent by using Douglas fir (Pseudotsuga menziesii) bark, in which thermocompression under a high pressing temperature range of 200–300 °C was applied instead of steam treatment, and demonstrated that it was effective to manufacture binderless board under a higher temperature range to enhance the product quality without additional processing. However, it was determined that temperatures should be limited to within 200 °C in order to avoid darkening, which affects the surface appearance of boards.

Adding pulping effluent as an adhesive not only simplifies the fabrication process, but also drives the cost down during fiberboard production [23]. Pulping effluent is always produced in the process of fiber manufacturing and papermaking, and is a complex mixture of inorganic and organic matter mainly containing lignin and polysaccharide [24]. A substantial amount of wastewater also needs to be treated in this process, since the water causes high chemical oxygen demand (COD) and becomes dark in color, with lignin and phenolic substances present [25]. However, these elements are critical for adhesion between fibers [26]. Thereby, the valorization of wheat straw and pulping effluent as a binder could utilize the resources efficiently and contribute to the worldwide zero waste strategy. Currently, effluent is still underutilized due to its inherent complexity and inefficiency [27], which makes it difficult to be recycled for industrial production. The bonding mechanism of straw-based fiberboards with pulping effluent therefore requires further study. In our previous work [23], the properties of the boards with variable amounts of pulping effluent were investigated, but gaps in knowledge remain concerning the effect of temperature towards the properties of straw-based fiberboards without adhesive but with the addition of pulping effluent.

Here, this study aims to manufacture fiberboards using wheat straw by adding pulping effluent instead of synthetic binders with different hot-pressing temperatures, and to investigate the effect of temperature on the properties of straw-based fiberboards. 

## 2. Materials and Methods

### 2.1. Materials

The wheat straw, pulping fibers, and pulping effluent used in this study were supplied by Tianjin Rong Yeda Fiber Technology Co., Ltd., in Tianjin, China. The pulp was passed through a defibrator by alkaline, and the straw and pulp were air-dried for three days at room temperature before testing. Pulping effluent was dried by lyophilization (LJC-10C; Four-ring, Beijing, China) for 48 h. 

### 2.2. Main Chemical Components

Wheat straw and pulp fibers were milled into 40–60 mesh of powder by a plant mill. The cellulose [28], ash [29], extractive [30,31,32], and Klason lignin [33] content were then determined. The pH of the pulping effluent was measured by a LEICI PHS-3C (LEICI, Shanghai, China) and a Shimadzu UV-2600 spectrometer (Shimadzu, Kyoto, Japan) was used to determine the content of protein according to the method of Waddel [34].

### 2.3. Straw-Based Fiberboard Production

The pulp fibers were mixed with 10 wt.% pulping effluent. The boards were shaped from the mixture manually using a forming box (300.0 mm × 300.0 mm), with a target thickness of 1.5 mm and a target density of 1000 kg/m^3^. After the boards were shaped, the mats were dried to 30% moisture content and pressed at a pressure of 7.5 MPa for 4 min under different temperatures (160, 170, 180, 190, and 200 °C) in triplicates. Meanwhile, three panels without pulping effluent were produced under 180 °C as the blank control group. After the thermomechanical process, all specimens were stored in a conditioning chamber at 20 °C with a relative humidity of 65% to reach an equilibrium state.

### 2.4. Fourier Transform Infrared (FT-IR)

The Fourier transform infrared (FT-IR) analyses were conducted using a Nicolet Spectrometer IS10 (Nicolet, Madison, WI, USA) to identify the structural changes of the functional groups under a variable hot-pressing temperature. Prior to the analysis, the specimens were ground and mixed with potassium bromide (KBr). All spectra were collected in the wave number range between 3000 and 400 cm^−1^, with dots per inch (DPI) of 4 cm^−1^ and at least 32 scans of each specimen.

### 2.5. Thermo Gravimetric Analyses (TGA)

The wheat straw and boards were processed by thermo gravimetric analyses (TGA). Thermal transitions were measured by TA Instruments Q100 TGA (TA Instruments, New Castle, DE, USA), at a heating rate of 10 °C/min under nitrogen with a 20 mL/min flow rate. The samples were heated from ambient temperature to 750 °C.

### 2.6. Physical and Mechanical Characterization

The modulus of elasticity (MOE), modulus of rupture (MOR), water absorption (WA), thickness swelling (TS), and internal bonding strength (IB) of the fiberboards were measured for each specimen, according to Chinese standards [35]. MOE and MOR were measured by three-point static bending tests on specimens with dimensions of 150.0 mm × 50.0 mm × 1.5 mm at a crosshead speed of 5 mm/min using Instron 5582 (Instron, Grove, PA, USA). IB was also measured by pulling the specimens (50.0 mm × 50.0 mm × 1.5 mm) apart in a perpendicular direction using Instron 5582. The mass and thickness of specimens with dimensions of 50.0 mm × 50.0 mm × 1.5 mm were measured before and immediately after the 24 h soaking process at 20 °C, and WA and TS refer to the percentage increase in the mass and thickness of a specimen, respectively. Each operation was performed in six replicates, and the mean value with standard deviation (SD) was calculated. A one-way analysis of variance (ANOVA) was applied to examine the effects of hot-pressing temperature on the properties of the boards. A Tukey’s test (α = 0.05) was used to determine which groups were significantly different from one another. All statistical analyses were operated using IBM SPSS Statistical Version 22.0 (IBM, Armonk, NY, USA).

## 3. Results and Discussion

### 3.1. Surface Appearance

**Figure 1:**
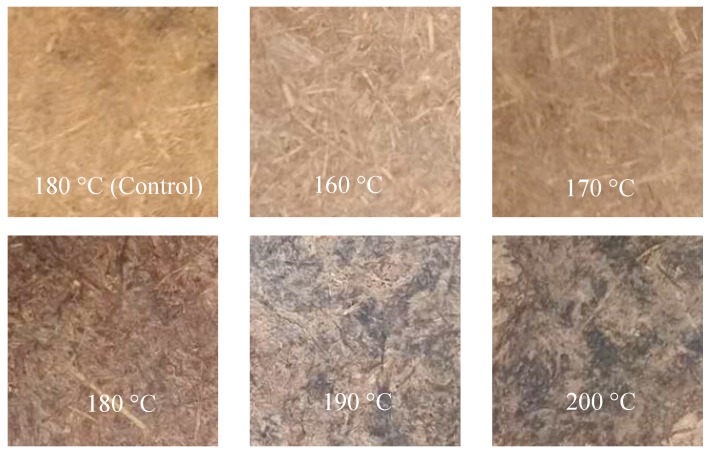
Photographs of fiberboards produced at different temperatures.

The manufactured fiberboards exhibited smooth surfaces [36], and the degree of surface darkening (Figure 1) with evaluated treatment temperature corresponded well with previous research [37]. This benefited primarily from the chemical modification of the wood constituents and the transference of the extractives [37]. According to other studies, the intensity of thermal degradation was directly related to the intensity of surface darkening, and was explained by hemicellulose hydrolysis and an increase in acid-insoluble lignin [38]. Similar fiberboards have been produced from unripe coconut husks, with high levels of hydrolysis in the biomass and the modification of chemical components in the process of hot pressing [39].

### 3.2. Chemical Characterization

Previous studies have demonstrated that lignin, as well as other carbohydrate substances, i.e., sugar and starch, are potential chemical constituents contributing to the bonding of fibers [16,40]. The chemical compositions of wheat straw and pulp are listed in Table 1. The wheat straw contains abundant α-cellulose (55.68%), providing several valorization opportunities in preparing fiberboards. In our previous work [23], the chemical composition of pulp fibers revealed moderate lignin and low ash contents. As a result, wheat straw could be used as an alternative low-cost material to manufacture fiberboards paralleling those acquired using milled wood according to Migneault et al. [41] and Mancera et al. [42]. In the comparison of wood resources, straw contains a smaller amount of additional secondary components, such as extractives, in addition to cellulose, hemicellulose, and lignin. Analogous results have also reported the use of other agricultural materials to create fiberboards without supplement, such as white coir and pith (α-cellulose: 27.0%, 23.0%; Klason lignin: 38.7%, 41.1%) [39]. Additionally, the extractives generated by tannins, oligosaccharides, and aromatics are potentially rich in pulping fiber, and conducive to forming phenolic bridges between lignin and carbohydrates (lignin–carbohydrate complexes (LCCs)). These bonds could enhance the mechanical capacities of straw-based boards with mechanical entanglement through the softened lignin molecules under hot pressing. Lignin and protein in the pulping effluent (Table 2) had a positive effect on the mechanical properties of boards, as demonstrated in previous studies concerning the application of lignin and proteins in adhesive formulas [43,44,45]. Furthermore, the formulation with 30 wt.% black liquor mostly conformed to the requirements of Chinese standards for medium density fiberboards. Due to the glass transition temperature of around 90 °C and the melting point of approximately 170 °C [46], lignin should be considered a thermoplastic material that becomes soft in the presence of heat and humidity [44]. Consequently, pressing temperature is a key parameter in the preparation process.

### 3.3. Imaging FT-IR Spectra

**Figure 2:**
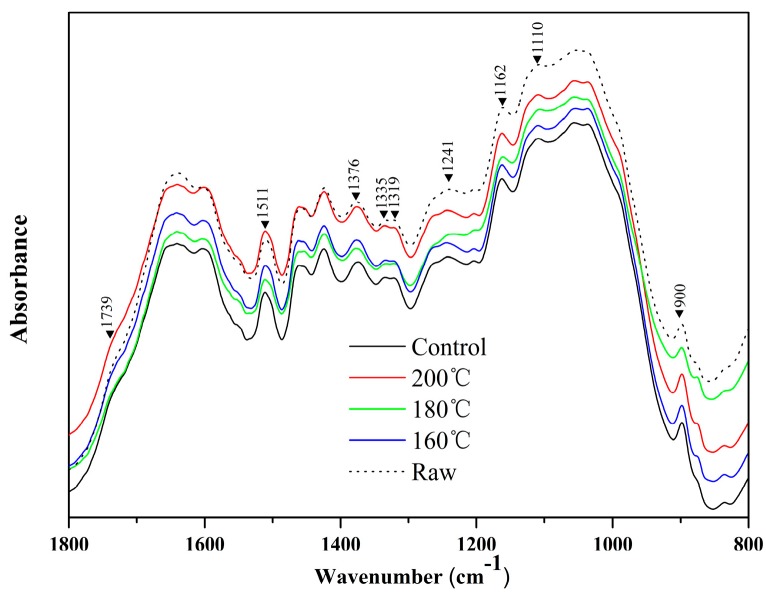
Fourier transform infrared (FT-IR) spectra of wheat straw and fiberboards produced at different temperatures.

Functional group analysis of the FT-IR spectrogram was based on the work of George and Mclntyre [47]. The FT-IR spectra bands were predominantly assigned to cellulose, hemicellulose, and lignin, which are the major components of straw wheat. The IR spectra of the raw materials, controlled fiberboards, and boards with effluent under different hot-pressing temperatures are illustrated in Figure 2. The results of the FT-IR showed two special sections (not presented) that could be identified: (i) the functional section in the 3800–2700 cm^−1^ range, assigned to different C–H stretching vibration groups and O–H stretching absorption [48], and (ii) the fingerprint section, from 1800–800 cm^−1^, assigned to different stretching or bending vibrations of the functional groups of biomass components. After hot-pressing treatment, several significant changes in peak intensity could be observed in the spectra of fiberboards at 1739, 1376, 1335, 1319, 1241, 1162, 1110, and 900 cm^−1^. Absorbance bands with the wave numbers of 1000–1200 cm^−1^ were characteristic of C–O vibration of cellulose and xylan. With the increase of temperature, there was a shift of the C–O carbonyl band at 1739 cm^−1^ and the C–O absorption band at 1241cm^−1^. This finding indicated that more hemicellulose was hydrolyzing, and that there was an increase in the hydrolysis of aldehyde compounds such as hydroxymethylfurfural and furfural. Recently, some researches have demonstrated that the characteristics of lignin as a plastic material could improve the mechanical performance of binder-free fiberboards [19,49,50,51]. The initial lignin content of the boards at different pressing temperatures were mostly identical to that shown in Table 1; as such, it was reasonable to assume it would display almost the same peak intensity at 1511 cm^−1^, derived from the aromatic units in lignin. Consequently, the peak at 1511 cm^−1^ was used as a constant standard to derive the relative absorbance of fiberboards with effluent, which differed from the boards without effluent and raw materials. Furthermore, the spectra at 1376, 1162, and 900 cm^−1^ were more significant under higher hot-pressing temperatures. The change at 1162 cm^−1^ was supposed to generate new ether linkages resulting from the reaction between hemicellulose by-products and lignin during the hot-pressing process, which was the lignin–carbohydrate complex (LCC). The increased intensity at 1376 and 900 cm^−1^ could be attributed to the polymerization of glycosidic components among low-molecular carbohydrates [52]. These linkages could enhance the self-bonding strength of boards without adhesion. The effluent also contained proteins, as shown in Table 2. The main vibration bands of the proteins were at 3300, 3100, 1690–1600, 1575–1480, 1301–1229, 767–625, 800–640, 606–537, and 200 cm^−1^. However, the typical vibration bands were not identified in overlapping sludge spectra [53]. Overall, the differences among the FT-IR spectra of samples at varying pressing temperatures were not significant. This result suggested that the chemical changes that occurred were either few or infrared-inactive during the hot-pressing process. 

### 3.4. Thermal Stability

**Figure 3:**
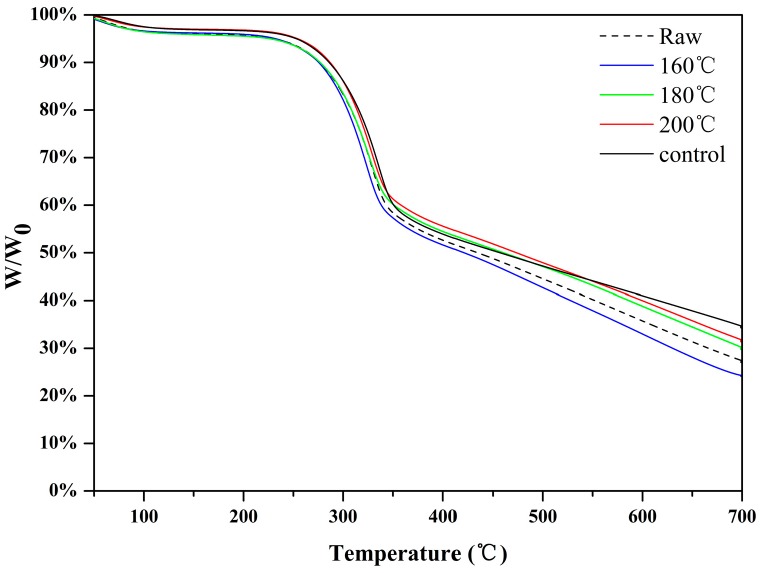
Pyrolysis curves of wheat straw and fiberboards produced at different temperatures.

The thermogravimetric properties of the blank control group and different board types under diverse hot-pressing temperatures are provided in Figure 3 as mass loss curves. Table 3 presents a selection of the specimen thermal properties, where each specimen was measured twice. The thermal behavior of fiberboards is relevant in the determination of process conditions for binderless board production. The curves depict three steps, and the main mass loss was between temperature levels of 200 and 300 °C. This mass loss could be due to the thermal decomposition of water-soluble components, hemicellulose, and a portion of cellulose. The board panels under 200 °C had the highest temperature for the mass loss of 10%, and lowest mass loss compared to boards with pulping effluent. Approximately 52.79% of samples pressed at 200 °C were degraded at a temperature below 500 °C, which was the least among all specimens. This finding was also supported by Lee et al. [54], who found that the minimum thermal decomposition rates for major sugars, including sucrose, glucose, and fructose, were at 138, 150, and 107 °C, respectively. There were two factors contributing to this trend. The first factor was the fragmentation of polysaccharides [55]; it was well understood that the removal of extractives and the fragmentation of hemicellulose and lignin all took place at high temperatures, and this result also corresponded well with the FT-IR analysis. On the other hand, the thermomechanical process, along with steam, was caused by the 30% moisture content of the slab as well as the presence of OH groups between fiber interfaces. In addition, the temperature for 10% mass loss of control boards was slightly higher than that of boards with effluent, and the total mass loss was reduced. As a possible reason for this outcome, it could be presumed that thermal softening of lignin played an important role in board performance expression. Thermal softening changes could involve glass transition because the local temperature of the board during hot pressing and the local glass transition temperature (*T*-*T*_g_) value were reported to correlate to the mechanical performance of the boards [56,57].

### 3.5. Physical-Technological Properties of Fiberboards

Table 4 describes the mean values and standard deviations of physical-technological properties, using ANOVA and Tukey’s test results. Values followed by identical lowercase superscripted letters (a, b, and c) indicate no statistical differences (*p* > 0.05). The MOR, MOE, TS, WA, and IB of the straw-based binderless fiberboards were improved by adding effluent, in comparison with the control fiberboard. Similar improvements in mechanical properties due to the treatment process were observed in a previous study [23]. However, the difference of this study from others was that boards with different densities were produced and the densification produces enhanced the board mechanical capacities. The results (Table 4) in this study showed that the mechanical properties of fiberboard samples were significantly influenced by the pressing temperature (*p* < 0.01). The Tukey’s test indicated that there was no remarkable difference in the physical-technological properties when increasing the temperature from 160 to 170 °C, except the TS. Nevertheless, there was a statistically significant difference in the range of 180 to 200 °C. The TS and WA of the boards decreased with an increase of the pressing temperature, reaching a minimum value (28.0% and 80.2%, respectively) at 200 °C (Table 4). This tendency was expected since high temperatures promoted the exudation of extractives, as well as a more hydrophobic surface of boards [58]. The boards pressed at 190 °C exhibited the highest MOR of 20.16 MPa, the highest MOE of 2994 MPa, and the higher IB of 1.02 MPa. The MOR, MOE, and IB of boards at 190 °C were 56.60%, 71.38%, and 29.11%, respectively, higher than those obtained at 160 °C. Therefore, binderless fiberboards from wheat straws and pulping effluent were found to reach the Chinese standard GB/T 21723 [59], except for the TS (MOR ≥ 14 MPa, MOE ≥ 1800 MPa, IB ≥ 0.45 MPa). Similar straw-based boards using steam explosion were produced by Luo et al. [60], and their experiments verified that binderless spruce and pine fiberboards had better water resistance than those made from wheat straw. The straw-based binderless fiberboards are not the final product, however, and can be further utilized after coating with a waterproof membrane. In such a case, the products would be used in normal climate conditions. In addition to temperature, it is suggested that one possible way to further improve adhesion is to chemically activate lignin in the effluent, by using enzymatic hydrolysis treatment so that the binding between fibers can be promoted during hot pressing. 

## 4. Conclusions

This work investigated the properties of manufactured straw-based fiberboards without resin under different hot-pressing temperatures. The chemical composition of pulp revealed moderate lignin and low ash contents, which could be used as an alternative low-cost source to manufacture fiberboards. Fiberboards without synthetic adhesive were prepared by hot-pressing mixtures of pulp and effluent at 160–200 °C. The pulping effluent content was tested, and the mechanical performance of boards pressed under various temperatures was determined. The FT-IR curves suggested that the proteins and lignin in the effluent were beneficial in creating new covalent bands under higher thermal-pressing temperatures, and thus showed better MOR, TS, and IB performances. Nevertheless, with a pressing temperature higher than 190 °C, the color of the boards became dark and there was excess energy consumption. There was no significant difference at 160–170 °C, or when the pressing temperature reached up to 190 °C. The properties of the straw-based fiberboards were improved significantly, illustrating that pulping effluent can be used as an adhesive. It is suggested that further studies be conducted in relation to activating lignin in effluent water before this technology can be implemented commercially.

## Figures and Tables

**Table 1:** Chemical compositions of wheat straw and pulp fibers.

Characteristics	Ash (%)	α-Cellulose (%)	Holocellulose (%)	Lignin (%)	Extractives (%)			
					Cold Water	Hot Water	1% NaOH	Alcohol–Benzene
Wheat straw	8.32	55.68	60.00	12.80	14.22	15.84	37.84	1.49
Pulping fibers	5.97	35.71	36.75	11.18	40.78	49.54	51.43	3.97

**Table 2:** Chemical composition of pulping effluent.

Characteristics	pH	Density (g/mL)	Dissolved Solids Concentration (g/L)	Extractive (Alcohol–Benzene) (%)	Ash (mg/mL)	Lignin (mg/mL)	Protein (mg/mL)
Values	9.17	1.00	17.50	0.09	752	190	2.95

**Table 3:** Thermal properties of wheat straw and fiberboards produced at different temperatures.

Specimens	Onset Mass (mg)	Temperature for the Mass Loss of 10% (°C)	Mass Loss at *T* ≤ 500 °C (%)	Final Mass (mg)	Total Mass Loss (%)
Raw	8.38669	270.34	55.96	2.26536	72.99
160 °C	6.59972	271.31	59.23	1.59229	75.87
180 °C	8.92963	270.01	53.40	2.64878	70.34
200 °C	8.61852	277.82	52.79	2.68206	68.88
Control (180 °C)	13.75989	277.32	53.33	4.69269	65.90

**Table 4:** The mean values and standard deviations of physical-technological properties, obtained using one-way analysis of variance (ANOVA) and Tukey’s test results.

Groups	Modulus of Rupture (MOR) (MPa)	Modulus of Elasticity MOE (MPa)	Thickness Swelling (TS) (%)	Water Absorption (WA) (%)	Internal Bonding Strength (IB) (MPa)
Control (180 °C)	16.4 ± 2.0	1914 ± 158	33.3 ± 3.2	95.0 ± 5.1	0.84 ± 0.07
160 °C	15.9 ± 1.5 ^b^	1747 ± 124 ^b^	33.9 ± 2.7 ^a^	99.1 ± 4.7 ^a^	0.79 ± 0.04 ^c^
170 °C	17.5 ± 1.2 ^b^	2046 ± 480 ^b^	32.8 ± 1.5 ^ab^	95.3 ± 5.9 ^a^	0.81 ± 0.06 ^c^
180 °C	21.2 ± 1.6 ^b^	2892 ± 207 ^a^	31.1 ± 1.0 ^abc^	91.4 ± 6.4 ^ab^	0.90 ± 0.03 ^bc^
190 °C	24.9 ± 0.9 ^a^	2994 ± 554 ^a^	29.5 ± 2.4 ^bc^	86.5 ± 2.7 ^bc^	1.02 ± 0.11 ^ab^
200 °C	24.0 ± 1.4 ^a^	2907 ± 292 ^a^	28.0 ± 2.6 ^c^	80.2 ± 4.2^c^	1.11 ± 0.08 ^a^
*p*-level	0.000 *	0.000 *	0.000 *	0.000 *	0.000 *
Straw-based wood [60]	18.10	–	–	63.7	0.24
Spruce and pine residues [16]	22.5	5000	18	45	0.75

Different uppercase letters in each row and lowercase letters in each column depict statistical differences (*p* < 0.05). “*” means significantly different at *p* < 0.01.
